# Supplementary material for: Mutations in PCYT2 disrupt etherlipid biosynthesis and cause a complex hereditary spastic paraplegia
Source: Brain. 2019 Oct 22;142(11):3382–97. doi: 10.1093/brain/awz291 (PMC6821184; doi:10.1093/brain/awz291)
Supplement: awz291_Supplementary_Data [file awz291_supplementary_data.zip › awz291-Suppl_data/Supplementary_Data4.zip!/Supplementary information 4 - plasma lipidomics.zip!/Plasma lipidomics/Multiple comparison/PLS-DA_alternative.pdf]

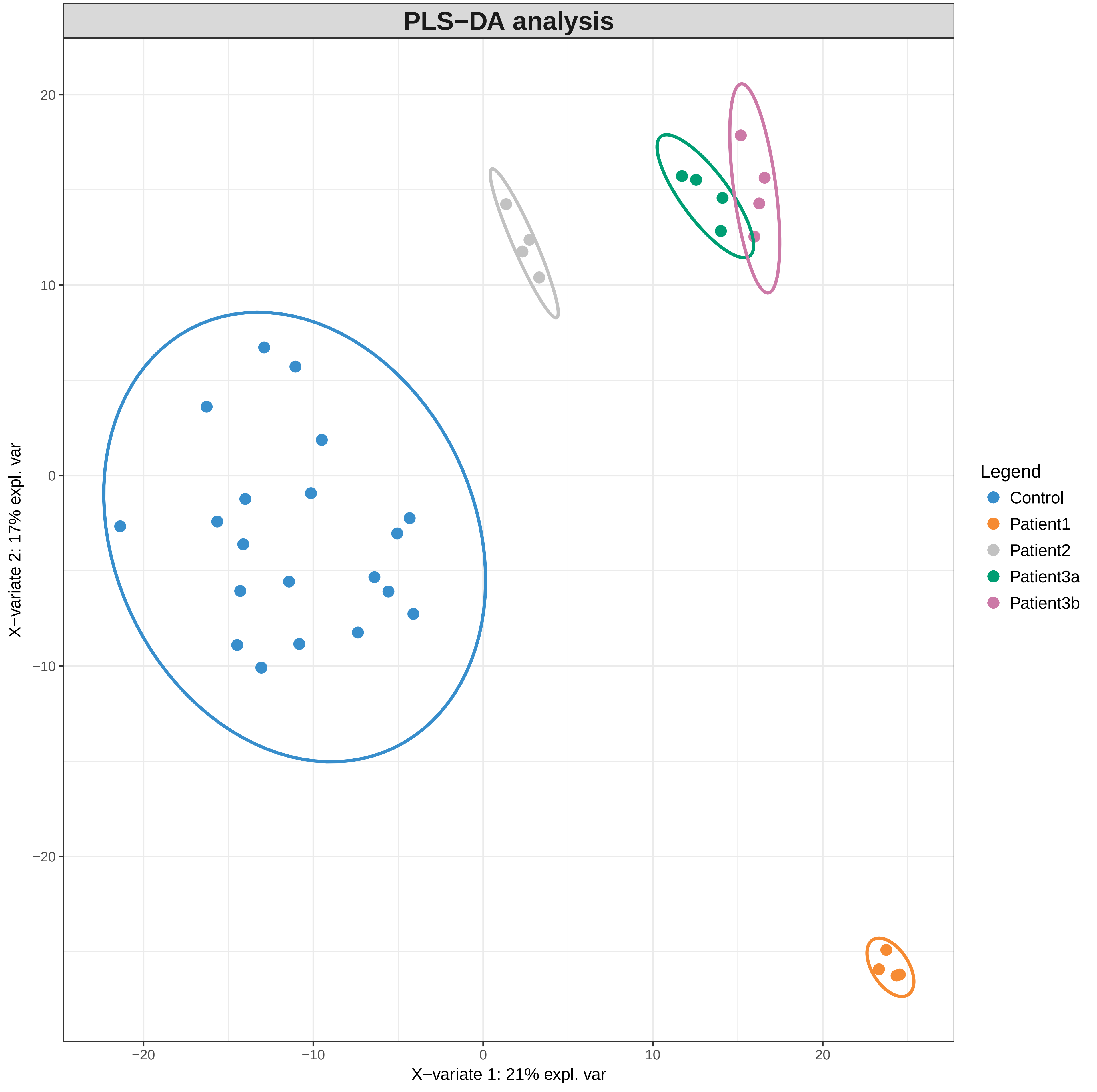

PLS−DA analysis
20
10
0
−10
−20
X−variate 2: 17% expl. var
−20
−10
0
10
20
X−variate 1: 21% expl. var
Legend
Control
Patient1
Patient2
Patient3a
Patient3b
